# Genetic insights and prospects of Egyptian- Nubian (Zaraibi) goats

**DOI:** 10.1007/s11250-025-04433-4

**Published:** 2025-05-08

**Authors:** A. M. Aboul-Naga, Hamdy Abdel-Shafy, Shaimaa A. Mohamed, Rasha M. Ahmed

**Affiliations:** 1 Animal Production Research Institute, Agriculture Research Center, Dokki, Cairo, Egypt; 2 Department of Animal Production, Faculty of Agriculture, Cairo University, Giza, Egypt

**Keywords:** Zaraibi goats, Genetic improvement, Subtropical goats, Candidate genes, Opportunities and prospects

## Abstract

The Egyptian Nubian (Zaraibi) goat has significant potential to contribute to animal production and rural livelihoods in Egypt. This article explores the breed’s unique genetic makeup and potential for improvement through examining its genetic parameters, population structure, genetic diversity, and candidate genes associated with economically important traits. The reported potential candidate genes include *caseins, alpha-lactalbumin (α-LA), beta-lactoglobulin (β-LG), prolactin receptor (PRLR), Fec* gene*, GDF9*, and *growth hormone*. These genes are associated with milk yield and composition, litter size, and growth performance. Although most candidate gene studies on Zaraibi goats have limitations, their findings can be utilized in genomic evaluation to improve perdition accuracy. Despite challenges such as small herd size, experimental design constraints, epigenetic influence, and potential trade-off between productivity and heat tolerance, there are significant opportunities to enhance the breed’s productivity and resilience. The studies highlight advantages such as high genetic diversity, positive genetic gain, adaptation to hot dry environment and clear genetic distinction from other local goat breeds. Prospects for improving Zaraibi goats include recognizing their socioeconomic role in rural communities, supporting selective breeding programs, integrating genomic information into selection strategies, implementing an open nucleus breeding scheme, and expanding the specialized goat cheese market. Like other promising subtropical breeds, utilizing their potential while addressing existing challenges is essential for ensuring sustainable production and continued contributions to the rural economy.

## Introduction

**Fig. 1:**
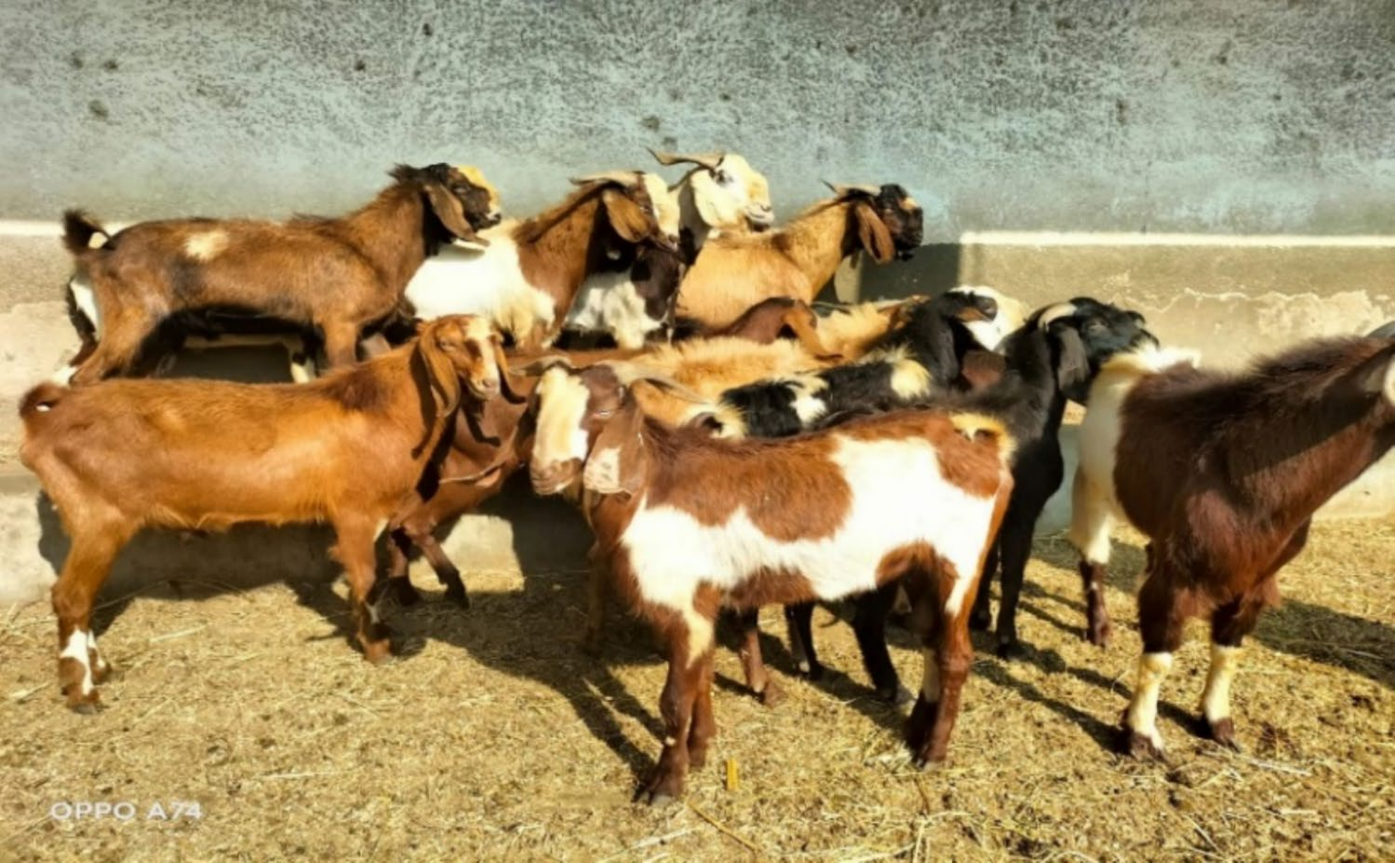
Zaraibi goat herd

The Egyptian Nubian (E. Nubian) goat, named after the Nubba region southern Egypt, where it was first recognized, is also referred to as “Zaraibi”. They bred as a dairy animal in confinement, at peri-urban areas (Zaraba in Arabic). The E. Nubian goat is considered one of the progenitors of the standard Anglo-Nubian goats (Devendra, 1982). The breed suited well with the subtropical climates, and is known for moderate milk yield, frequent multiple births, and good growth performance (Galal et al. 2005; Aboul-Naga et al. 2012)**.** Distinctive physical features of the breed include a convex profile, Roman nose, common undershot jaw, good body size, long legs, and dropping pendulum ears. Common coat colors are brown, white, or dark black (Fig. 1).

Early in the eighties, Animal Production Research Institute (APRI), Egyptian Ministry of Agriculture and Land Reclamation, initiated a successful trial to establish a nucleus herd of E. Nubian goats, with aims of characterizing, preserving, and improving them. The project led to significant achievements including characterization of the breed’s genetic makeup and the established a long-term conventional selection program to improve their milk production and growth rate while maintaining their high prolificacy (Hamed 2010; Rasha 2020). These efforts have been instrumental in preventing their genetic erosion and promoting the sustainability of the breed.

Conventional breeding programs based on phenotypic information are inherently slow; and particularly challenging for local breeds, with limited pedigree information and small herd sizes, which can hinder significant genetic progress. Furthermore, phenotypic selection alone may not effectively capture the complex genetic architecture of the complex traits, such as tolerance to heat stress and fertility, which are crucial for the breed’s adaptation and productivity. Therefore, integration of genomic information into genetic evaluation and selection decision would be a good alternative. In this regard, the recent methods, such as genome-wide association studies (GWAS), candidate gene approaches, and genomic selection; offer powerful tools to identify genomic loci associated with trait variation more accurately and consequently could accelerate its genetic gain. These methods provide more precise and efficient alternative to traditional breeding, enabling rapid improvement and sustainable development of local breeds (Abdel-Shafy 2021). Meanwhile, genetic, and genomic information for local subtropical goat breeds is limited. Recent studies have begun to shed light on the genetic characterization and diversity of local goat breeds. For instance, GWAS analysis of E. Nubian goats has revealed distinct clusters from other local breeds (Aboul-Naga et al. 2023). This finding highlights the importance of further genetic research to fully understand the unique genetic makeup of the local breeds including E. Nubian goat.

The current article aims to compile existing genetic, genomic, and breeding information on the E. Nubian goat; highlighting genetic parameters, associated genetic markers for economical traits, and explore their phylogenetic relationships. In addition, the article discusses the challenges and opportunities in their breeding programs and offers perspectives for improving such important subtropical goat breed.

## Genetic parameters of E. Nubian (Zaraibi) goat

Genetic parameters including heritability, repeatability, and genetic correlation describe the genetic variation within a population for specific traits. Heritability estimates for productive and reproductive traits of the Zaraibi goat ranged from low to moderate (Table 1, 2 and 3). The estimates vary between populations and changes in management and environmental conditions. They also depend on the number of records available and method of estimation.

**Table 1:** Heritability estimates for growth traits of Zaraibi kids

No of animals	Method	BW**	W2M	W4M	W12M	Ref
352	HN *	0.29		0.12		Bata (1989)
4707	RR	0.37		0.28	0.53	Mekkawy (2000)
6755	DFREML	0.21			0.12	Shaat et al. (2007)
6610	AI-REML	0.08—0.24				Shaat and Mäki‐Tanila (2009)
10,374	MTDFREML	0.19	0.13			(Aboul-Naga et al. 2012)
10,374	MTDFREML	0.13- 0.29		0.11—0.22		Mona (2013)
2159	MTDFREML; HN1; ML; MV	0.16 ± 0.030- 0.25 ± 0.087		0.19 ± 0.054- 0.26 ± 0.080		El-Moghazy et al. (2015)
2998	MTDFREML	0.23—0.43	0.20—0.31	0.17–0.34		El-Awady et al. (2019)
13,062	MTDFREML			0.28	0.38	(Rasha et al. 2020)

*MTDFREML Multi-Traits Derivative-Free Restricted Maximum Likelihood, *HN* Henderson’s method I, *ML* Maximum Likelihood, *MV* Minimum Variance Quadratic Unbiased Estimation, *AI-REML* Average information REML, *DFREML* Derivative-Free Restricted Maximum Likelihood, *RR* random regression **BW birth weight, *WW* weaning weight, *W2M* weight at 2 months, *W4M* weight at 4 months, *W12M* weight at 12 months

**Table 2:** Heritability estimates for reproduction traits of Zaraibi kids

No	Method	LSB**	LSW	LWB	LWW	Reference
592	MTDFREML*		0.02	0.03	0.01	Abdel-Raheem (1998)
820	MTDFREML	0.18 ± 0.05 0.22 ± 0.08	0.15 ± 07	0.09.06 0.16 ± 0.04	0.06 ± 0.05 0.14 ± 0.07	Farrag et al. (2007)
1380	AI-REML algorithm	0.04 ± 0.02 0.05 ± 0.04				Shaat and Mäki‐Tanila (2009)
2202	MTDFREML	0.08	0.05			Aboul-Naga et al. (2012)

*MTDFREML Multi-Traits Derivative-Free Restricted Maximum Likelihood, *AI-REML* Average information REML

**LSB Litter size at birth, *LSW* Litter size at weaning, *LWB* Litter weight at birth, *LWW* Litter weight at weaning

**Table 3:** Heritability estimates for milk production traits of Zaraibi goats

NO	Method	Total milk yield	Lactation length	Ref
592	MTDFREML*	0.10	0.03	Abdel-Raheem (1998)
820	MTDFREML	0.24 ± 0.10	0.12 ± 0.09	Farrag et al. (2007)
975	MTDFREML	0.35	0.15	Shaat et al. (2007)
1380	AI-REML	0.23 ± 0.05 - 0.24 ± 0.02		Shaat and Mäki‐Tanila (2009)
1815	MTDFREML	0.26		Aboul-Naga et al. (2012)

*MTDFREML Multi-Traits Derivative-Free Restricted Maximum Likelihood, *AI-REML*: Average information REML

Heritability estimates for body weight at different growth stages of Zaraibi goats range between 0.16 and 0.30, with few exceptions. For instance, Bata (1989) estimated heritability for birth weight (BW) as 0.29 and weaning weight (WW) as 0.12. Similarly, Mekkawy (2000) reported high estimates of 0.37 for birth weight (BW), 0.28 for WW and 0.53 for W12M. Shaat et al. (2007) provided estimates of 0.21 for BW and 0.12 for weight at 12 months (W12M). Aboul-Naga et al. (2012) and Mona (2013) reported estimates of 0.19 to 0.29 for BW and 0.13 to 0.22 for W12M. El-Moghazy et al. (2015) and El-Awady et al. (2019) reported similar estimates. These results suggest a good positive response to selection for body weight, especially at early stages at birth and weaning. The high standard deviations in some studies reflect poor estimations, mostly due to small sample size.

For reproductive traits, heritability estimates were generally low, ranging from 0.04 to 0.15. Shaat and Mäki‐Tanila (2009) reported even lower estimates as 0.04 ± 0.02 for litter size at birth, whereas Farrag et al. (2007) reported moderate estimate of 0.22 for the same trait. Generally, the reported estimates indicating limited potential for genetic improvement of the reproductive traits in the Zaraibi goats.

Heritability estimates for milk yield are moderate, ranging from 0.10 to 0.24. For instance, Abdel-Raheem (1998) reported heritability of 0.10 for total milk yield and only 0.03 for lactation length. Farrag et al. (2007) and Shaat et al. (2007) found higher estimates of 0.24 and 0.35 for total milk yield, respectively. These moderate values suggest good positive response to selection for milk yield.

Genetic correlations among economical traits have been confirmed, indicating that selection for one trait can impact the others (Table 4). For instance, Shaat et al. (2007) reported genetic correlation of 0.42 between BW and 3 months weight (W3M), as well as 0.62 between BW and W12M. Correlation between W3M and W6Mwas 0.77 while with W12M it was 0.82. Genetic correlation between total milk yield and 90-day milk yield was 0.89, and was 0.80 with lactation length. Farrag et al. (2007) estimated genetic correlations between kids born and litter weight at birth (0.52) and weaning (0.61), as well as between litter weight at weaning and total milk yield (0.71). Shaat and Mäki‐Tanila (2009) also reported significant correlation between litter size and 90-day milk yield (0.45). These positive correlations suggest that improving one trait would have beneficial effects on the other related traits.

**Table 4:** Genetic correlation between production traits in Zaraibi goat

Traits	#Record	Estimate	Ref
BW* – W3M	6755	0.42	Shaat et al. (2007)
-W12M		0.62	
W3M- W6M	6755	0.77	
- 12MW		0.82	
TMY- 90DMY	975	0.89	
-lactation length		0.80	
LSB—LWB	820	0.52	Farrag et al. (2007)
- LWW		0.61	
-TMY		0.21	
LWB -TMY	820	0.23	
-LWW		0.68	
LWW—TMY	820	0.71	
LS – 90DMY	1380	0.45	Shaat and Mäki‐Tanila (2009)
-TMY		0.22	
LSB- LSW	2202	0.91	Aboul-Naga et al. (2012)
- TMY		0.29	
LSW—TMY	1815	0.28	
W4M.—W6M	13,062	0.89	Rasha (2020)
W6M– W12M		0.92	

*LSB litter size at birth, BW birth weight, W3M W4M W6M weight at 3, 4 and 6 months, W12M weight at 12 months, LSW Litter size at weaning, LWB Litter weight at birth, LWW Litter weight at weaning, TMY total milk yield, 90DMY 90 days milk yield

Starting in 1983, a long-term conventional selection program was applied for the nucleus herd of APRI. The program covered both males and females, for economic traits such as milk yield at first parity, litter size and milk yield of the dams (for males). In 2004, the selection criteria were revised to include the breeding values of the animals for body weight at 4 and 12 months, and total milk yield at first parity. Bucks were selected based on their W12M and milk yield of the dams (Hamed 2010; Rasha 2020).

The selection program resulted in positive yearly gains for weaning and yearling weights, up to 300 gm/year (Aboul-Naga et al. 2012; El-Awady et al. 2019; Rasha 2020). Selection for milk yield led to encouraging results, with annual gains ranging from 220 to 720 gm of milk per year (Aboul-Naga et al. 2012; Rasha 2020). These figures highlight the effect of the selection program in enhancing growth and milk production in Zaraibi goats.

## Genetic characterization and associated markers for economically important traits

The genetic characterization of economically important traits in Zaraibi goats has been extensively studied using various molecular markers, including Restriction Fragment Length Polymorphism (RFLP), Microsatellites, Single Strand Conformational Polymorphism (SSCP), and DNA sequencing. These molecular tools facilitate the identification of genetic diversity and structure within the population, providing insights into the genetic basis of the key economic traits. In addition, it can be used to detect genomic regions and candidate genes associated. Such genetic insights are instrumental in enhancing selection programs, ultimately leading to significant improvements in productivity and sustainability of Zaraibi goats. This section reviews the key findings from these studies, highlighting genetic variation and specific markers associated with economic traits in Zaraibi goats.

### Milk production

Milk production and lactation period are affected by several factors including breed, litter size, parity, stage of lactation, and health conditions. Th**e** Zaraibi goats are considered the best milk producers among the Egyptian goat breeds**,** with reported milk yield of 249 to 363 kg/head/lactation (EL-Sayed et al. 2013; Abdelhamid et al. 2013). Studies in Zaraibi goats have identified several single nucleotide polymorphisms (SNPs) in candidate genes and tested their association with milk production traits (Table 5). However, the reliability of these associations depends upon the sample size and potential flaws in the experimental design. Specifically, some studies did not account for the genetic relationships, population structure or non-genetic factors in their statistical models, which might lead to potential false positive associations. Despite these limitations, these studies provide valuable insights for future research on genetic characterization, diversity analyses, and improving milk production in Zaraibi goats. Possible candidate genes reported for milk production are:

**Table 5:** Genetic markers related to milk traits in Egyptian Zaraibi goat

Gene	No of Animals	Methods	Genotypes& SNP	Genetic Association	Author
**CSN1S1**	10	RFLP**: (*XmnI*)	3 genotypes		Sahar (2006)
	165	RFLP *(XmnI)* & AS-PCR Seq.: exon 9, exon 15,16	9 genotypes & 15 SNPs	Milk yield*, Composition*	Teleb et al. (2016)
	50	SSCP &Seq	3 genotypes & 3 SNP	Milk Comp.*	Darwish et al. (2023)
**CSN1S2**	10	RFLP:*(MseI, PstI, NcoI, NlaIII and Alw26I)*	4 genotypes		Othman and Ahmed (2006)
	165	RFLP: *PstI*	1 genotype		Shaimaa (2016)
**K-CSN**	10	RFLP: *Alw44I* and *HaeIII*	3 alleles & 4 SNPs		Othman and Ahmed (2007)
	50	PCR-SSCP Seq	3 genotypes & 3 SNP	Milk Compos. *	Darwish et al. (2023)
**Alfa-lactalbumin**	10	PCR-SSCP Seq: exon 3	2 genotypes & 1 SNP	Milk yield*	Nowier et al. (2022)
**β-Lactoglobulin**	10	RFLP: *SacII and SmaI*	5 genotypes		Ahmed and Othman (2009)
**Diacyl Glycerol Acyl Transferase 1**	165	RFLP: *NlaIII* Seq.: exon 15,16	3 genotypes & 3 SNPs	Total Solids*	Eid et al. (2020)
	50	Seq.: exon 15,16	3 genotypes & 3 SNPs	Fat & Total Solids*	Mohamed et al. (2022)
**Prolactin**	20	RFLP:* Eco24I* Seq: exon 5	2 genotype, 1 SNP		Abdel-Aziem et al. (2018)
	20	PCR-SSCP	2 genotypes & 4 SNPs	Fat*	Darwish et al. (2023)
**Prolactin Receptor**	100	PCR-SSCP Seq.: 3’UTR	2 genotypes & 1 SNP	Milk yield*	El-Shorbagy et al. (2022)
	110	RFLP: *RsaI* & Seq.: 3’UTR	3 genotypes & 1 SNP	Milk yield* Litter Size^ns^	Nowier et al. (2023)
**Leptin**	100	PCR-SSCP Seq.: intron2, exon 3	Intron 2: 1 genotype & 1SNP		El-Shorbagy et al. (2022)
**Lactoferrin**	53	PCR-SSCP Seq: exon 2	2 genotypes &1 SNP	Protein, T. Solids & Solids nonfat*	Nowier et al. (2020)

* Significant at 5% & ns non-Significant, **RELP Restriction Fragment Length Polymorphism, PCR-SSCP Polymerase Chain Reaction-Single Strand Conformational Polymorphism, Seq Sequencing, AS-PCR Allele Specific PCR

- *Casein genes: *Casein is the main protein fraction of goat milk, valued for its nutritional and processing properties. Ferretti et al. (1990) reported that casein fractions are clustered within a 250-kb genomic DNA segment (from 85.978 to 86.211 Mb) on chromosome 6 of Capra hircus, arranged as CSN1S1, CSN2, CSN1S2 and CSN3. Sahar (2006) reported three genotypes for the CSN1S1 gene in Zaraibi goats, with BD being the most frequent genotype. Teleb et al. (2016) identified nine genotypes and 15 SNPs in this gene, linking the AD genotype to the highest daily milk yield (2.00 ± 0.18) and milk compositions including fat% (3.96 ± 0.47) and protein% (3.84 ± 0.35). Conversely, the DD genotype had the lowest milk yield (1.07 ± 0.09), while AA was associated with the lowest milk constituents. Furthermore, Othman and Ahmed (2006) demonstrated three genotypes and three SNPs in the CSN1S1, with AB genotype having the highest % of milk components. They also reported four genotypes for CSN2S2, with AB being the most frequent genotype. Shaimaa (2016) found one genotype for the CSN1S1. Additionally, Othman and Ahmed (2007) detected three genotypes and four SNPs for the CSN3, where the AG genotype was associated with the highest milk constituents.
- *Whey protein genes:* Alfa-lactalbumin (α-LA or LALBA) and beta-lactoglobulin (β-LG) constitute 87% of total whey proteins in milk (Acton 2013) and play important roles in nutrition, antioxidant activity, and immune defense (Marshall 2004). The *α-LA* gene is located on chromosome 5 of *Capra hircus*, spanning 2017 bp *(NCBI* Gene ID: 100,860,779), and is considered a candidate gene for milk yield and growth traits in farm animals. In Zaraibi goats, Nowier et al. (2022) sequenced exon 3, identifying two genotypes and one SNP associated with milk yield. Similarly, β-Lactoglobulin is involved in several biological processes, including prostaglandins synthesis, lipid digestion, passive immune transfer and antimicrobial against mastitis pathogens (Chaneton et al. 2011). The *β-LG* gene is situated in a GC-rich region on chromosome 11 of *Capra hircus* spanning 4711 bp (*NCBI* Gene ID: 108,637,095). Ahmed and Othman (2009) reported five genotypes related to milk production in Egyptian Nubian goats. Although very few studies have been performed on these genes, they provide valuable insights into the genetic characterization and diversity as well as potential for further studies in Zaraibi goats.
- *Diacylglycerol Acyltransferase1*: DGAT1 is a key enzyme in triacylglycerol synthesis and major contributor to genetic variation in milk production among dairy goats. The gene, located on chromosome 14 with 18 exons (Khan et al. 2021), has been studied for its association with milk composition. In this regard, Eid et al. (2020) detected three genotypes and three SNPs in the *DGAT1* gene, revealing associations with milk composition, particularly total solids. Similarly, Mohamed et al. (2022) identified three genotypes and three SNPs significantly linked to fat percentage and total solids.
- *Prolactin (PRL)*: The *PRL* gene plays a key role in growth, lactation, and in hair growth, influencing milk yield and composition (Rose et al. 1998). Abdel-Aziem et al. (2018) sequenced exon 5 of *PRL* gene, identifying two genotypes and one SNP in Zaraibi goats, Darwish et al. (2023) found significant associations between *PRL* genotypes and milk fat percentage, detecting two genotypes and four SNPs. The action of PRL is mediated by the *prolactin receptor* (*PRLR*) gene, a member of the *growth hormone/prolactin receptor* gene family (Ahlawat et al. 2016). In this regard, El-Shorbagy et al. (2022) observed two genotypes and one SNP for *PRLR* with significant effect on milk yield in Zaraibi goats. Likewise, Nowier et al. (2023) examined the same region, detecting three genotypes and one SNP significantly affecting milk yield.
- *Leptin (LEP*) and *Lactoferrin (LF*) genes: The *LEP* gene plays a central role in regulating animal productivity and body growth (Andziak et al. 2009). El-Shorbagy et al. (2022) reported one genotype and one SNP in intron 2 of the *LEP* gene, associated with milk production in Zaraibi goats, although its evaluation remains pending. The *LF* gene is a multifunctional gene involved in important traits such as milk protein composition and skeletal structure in goats (Ali and Al-Samarai 2018). Nowier et al. (2020) reported two genotypes and one SNP located in exon 2 of the *LF* gene in Zaraibi goat, with significant associations with milk composition.

### Reproductive traits

Reproductive performance is a key factor in the characterization and association studies of Zaraibi goat (Tesema et al. 2020). Despite their importance, studies on reproductive traits in Zaraibi goats are limited, primarily due to small sample sizes, which may lead to potential false-positive associations (Table 6). Additionally, many studies lack robust experimental designs that account for important factors such as population stratification and non-genetic influences. The following genes have been reported as potential candidates for reproductive traits in Zaraibi goats:

**Table 6:** Genetic markers related to reproductive performance for Egyptian Zaraibi goat

Methods	No	Methods	Genotypes& SNP	Genetic association	Author
Acrocin (Acr.)	10 ♂	Seq, exon 5	1 SNP		Jnied et al. (2013)
Kisspeptin	18	RFLP**: *XmnI* Seq.: Intron 1	2 genotypes & 1 SNP		Othman et al. (2015)
	44	RFLP: *XmnI* Seq.: intron 1	2 genotypes & 1 SNP	Litter Size*	El-Tarabany et al. (2017)
Bone Morphogenetic Protein, Receptor 1B (BMPR1B)	11	RFLP Seq	5 SNPs	Litter Size*	Helal et al. (2014)
Bone Morphogenetic Protein (BMP15)	15	RFLP: *HinfI* Seq.: exon 2	6 SNPs	Litter Size*	Heikal and El Naby (2017)
Boorola fecundity (FecB)	22	RFLP: *AvaII*	1 SNP		Othman et al. (2019)
G Protein-Coupled Receptor 54(GPR54)	22	RFLP: *TaqI* for exon 5, *HpaII* for exon 1	3 genotypes &1 SNP		Othman et al. (2019)
GDF9	40	T-ARMS-PCR	2 polymorphic sites	Fecundity*	Aboelhassan et al. (2021)
	40	RFLP: Msp1 for exon 1	2 genotypes & 2 SNP	Litter Size*	Metawi et al. (2021)
	100	Seq.: exon2 & qPCR-HRM	3 genotypes & 1 SNP	Litter Size*	Kotb et al. (2023)
Alpha- Lactalbumin	74	PCR-SSCP	2 genotypes & 1 SNP	Litter Size*	Nowier et al. (2022)
Prolactin Receptor	100	PCR-SSCP Seq.: exon 10	2 genotypes & 1 SNP	Litter size*	El-Shorbagy et al. (2022)
	110	RFLP: RsaI& Seq.: 3’UTR	3 genotypes & 1 SNP	Litter Size^ns^	Nowier et al. (2023)

* Significant at 5% & ns non-Significant**RELP Restriction Fragment Length Polymorphism, PCR-SSCP Polymerase Chain Reaction-Single Strand Conformational Polymorphism, Seq Sequencing, T-ARMS-PCR Tetra-primer amplification refractory mutation system PCR

- *Acrosine gene: *The acrosine gene encodes a multifunctional protein essential for fertilization, facilitating the recognition, binding, and penetration of the zona pellucida of the ovum (Klemm et al. 1991). This gene is located on chromosome 5 and consists of five exons. Jnied et al. (2013) identified only one SNP in exon 5 of Zaraibi bucks.
- *Kisspeptin* (*KISS1*) gene: The *kisspeptin* gene plays a crucial role in regulating the secretion of luteinizing hormone (LH) and follicle-stimulating hormone (FSH) in mammals (Dhillo et al. 2005). Othman et al. (2015) examined the *KISS1* gene in Zaraibi goats and detected two genotypes and one SNP. Similarly, El-Tarabany et al. (2017) reported genetic variants in the *KISS1* gene associated with litter size in Zaraibi goats.
- *TGF-β* Superfamily (*Fecundity* Genes): The *Transforming Growth Factor Beta* (*TGF-β*) superfamily, commonly known as fecundity (*Fec*) genes, plays an important role in regulating ovarian functions and embryo development. Key genes within this group, including *BMP15*, *BMPR1B* and *GDF9*, are associated with ovulation rate and litter size and overall reproductive efficiency in sheep and goats. *BMPR1B* is a dominant autosomal gene responsible for fecundity and twinning in sheep and goats (Davis 2004). It was first identified in Boorola Merino sheep as the gene responsible for high prolificacy associated with *FecB* mutation (Wilson et al. 2001). In Zaraibi goats, Helal et al. (2014) sequenced a 199 bp fragment of *BMPR- 1B* and detected five SNPs, which were significantly associated with litter size.
- *Bone morphogenetic protein (BMP15****)*** and *bone morphogenetic protein receptor- 1B (BMPR1B)* genes: *BMP15* is an oocyte-derived growth factor located on the X chromosome, playing a crucial role in ovarian development by regulating granulosa cell functions (Juengel and McNatty 2005). Polymorphisms in *BMP15* gene have been associated with improved reproductive traits in ruminants (McNatty et al. 2005). Heikal and El Naby (2017) found six SNPs in exon 2 of the *BMP15* gene, showing significant influence on litter size in Zaraibi goats.
- *Boorola fecundity* (*FecB*) gene: The *FecB* gene plays a major role in reproductive endocrinology, ovarian development, ovulation rate, and litter size (EL-Hanafy AA and El-Saadani MA 2009). In Zaraibi goats, Othman et al. (2019) revealed one SNP in the *FecB* gene.
- *Growth Differentiation Factor 9 (GDF9)*: *GDF9* gene is an important growth and differentiation factor involved in early folliculogenesis in female mammals. Several mutations of this gene, including *FecG*^*H*^***,**** FecG*^*E*^***,**** FecTT, G1*, and *G1111 A*, have been associated with fertility in sheep (Våge et al. 2013). Genetic polymorphisms in the *GDF9* gene have also been identified in Zaraibi goats. For instance, Othman et al. (2019) found two genotypes and one SNP in the exon 1 of this gene. Likewise, Metawi et al. (2021) detected two genotypes and two SNPs associated with fecundity in Egyptian goats. Aboelhassan et al. (2021) found two mutations with significant effects on fecundity in Zaraibi goats. Additionally, Kotb et al. (2023) revealed three genotypes and one SNP in exon 2 of the GDF9 gene. Notably, among different goat breeds, only Zaraibi goats exhibited two alleles (G and A), while other goat breeds possessed only the G allele Aboelhassan et al. (2021).
- *G protein-coupled receptor 54 (GPR54)* gene: *GPR54* is the endogenous receptor of the KISS1 peptide (Chu et al. 2012) and is highly expressed in the placenta, pancreas, and brain. It plays a key role in stimulating LH and FSH secretion to initiate puberty. In Zaraibi goats, only one study has been carried out, detecting three genotypes and one SNP in the *GPR54* gene (Othman et al. 2019).
- *Alpha-lactalbumin* (*α-LA*) gene: *α-LA* is a major whey protein with important roles in nutrition, growth, and development (Layman et al. 2018). Nowier et al. (2022) reported two genotypes and one SNP in exon 3 of the *α-LA* gene in Zaraibi goats, which significantly influnced litter size.
- *Prolactin Receptor (PRLR)* gene: *PRLR* is a strong candidate gene for analyzing QTL affecting litter size (Wu et al. 2014). By sequencing exon 10 of the *PRLR* gene in Zaraibi goats, El-Shorbagy et al. (2022) detected two genotypes and one SNP with significant effects on litter size. Similarly, Nowier et al. (2023) observed three genotypes and one SNP in the 3’UTR of this gene in Zaraibi goats.

### Growth traits

Similarly investigations on growth-related genes in Zaraibi goats faces limitations in its widespread application. The available studies are summarized in Table 7.

**Table 7:** Genetic Markers related to Growth traits for Egyptian Zaraibi goat

Gene	No Animals	Method	Genotypes& SNP	Genetic association	Author
**Growth** **Hormone**	20	RFLP**: *Haelll*	4 alleles		Alakilli et al. (2012)
	5	Seq.: exon 3	4 SNPs		El-Halawany et al. (2017)
	20	RFLP: *HaeIII* Seq.: Exon 2, 3& 4	GH1: 2 genotypes GH2: 1 genotype & 1 SNP & 2 gab		Mahrous et al. (2018)
	20	Seq.: exon 3	1 SNP	W4M, W6M and W12M) *	Rasha (2020)
	18	RFLP: *Hae III* 5’UTR, exon1, intron1 and exon 2	3 genotypes & 1 SNP		Abdelhafez et al. (2022)
**Insulin like growth factor I**	20	RFLP: *Hae III*	2 alleles		Alakilli et al. (2012)
**Pituitary specific transcription factor- 1**	20	RFLP: *Pst1*	2 alleles		Alakilli et al. (2012)
**Bone morphogenetic protein − 15**	20	RFLP: *Hinf1*	No variation		Alakilli et al. (2012)
**Myostatin**	20	RFLP: *Dra I*	2 alleles		Alakilli et al. (2012)
		Seq.: intron 1, exon 2	99% identity with Baladi		Dowidar et al. (2018)
**Alpha-** **Lactalbumin**	74	PCR-SSCP	2 genotypes & 1 SNP	Body Weight ^ns^	Nowier et al. (2022)

* Significant at 5% & ns non-Significant

**RELP Restriction Fragment Length Polymorphism, PCR-SSCP Polymerase Chain Reaction-Single Strand Conformational Polymorphism, Seq Sequencing, W4M weight at 4 months, W6M weight at 6 months, W12M weight at 12 months

- *Growth hormone (GH)* gene: GH is a pivotal gene with numerous physiological functions, influencing milk production, growth rate, and aging (Chung et al. 1998). Several genetic variations have been reported in the GH gene of Zaraibi goats, for instance, Alakilli et al. (2012) identified four alleles, while El-Halawany et al. (2017) observed four SNPs in exon 3. Mahrous et al. (2018) found two genotypes at GH1, along with one genotype, one SNP, and two gabs at GH2. Abdelhafez et al. (2022) recorded three genotypes and one SNP at the 5’UTR, exon 1 and exon 2 of the GH gene. Furthermore, Rasha (2020) found a significant association between one SNP at exon 3 and body weight at 4, 6, and 12 months in Zaraibi goats.
- *Insulin like growth factor I (IGF-I), Pituitary specific transcription factor- 1 (POU1 F1), Bone morphogenetic protein (BMP15) and Myostatin (MSTN)* genes: Several related genes, including *IGF-I*, *POU1 F1, BMP15,* and *MSTN,* have been characterized by Alakilli et al. (2012) in Zaraibi goats. They detected two alleles in *IGF-I, POUIF1,* and *MSTN* genes, while no variation was observed in the *BMP15* gene. Dowidar et al. (2018) sequenced intron 1 and exon 2 of the *MSTN* gene, revealing a 99% identity between Zaraibi and Baladi goats.
- *Alpha****-****Lactalbumin (LALBA)* gene: The *LALBA* gene has been associated with body weight in Zaraibi goats using two genotypes, one SNP detected in this gene, demonstrating its potential influence on growth traits (Nowier et al. 2022).

## Phylogenetic analysis of Zaraibi Goats

**Fig. 2:**
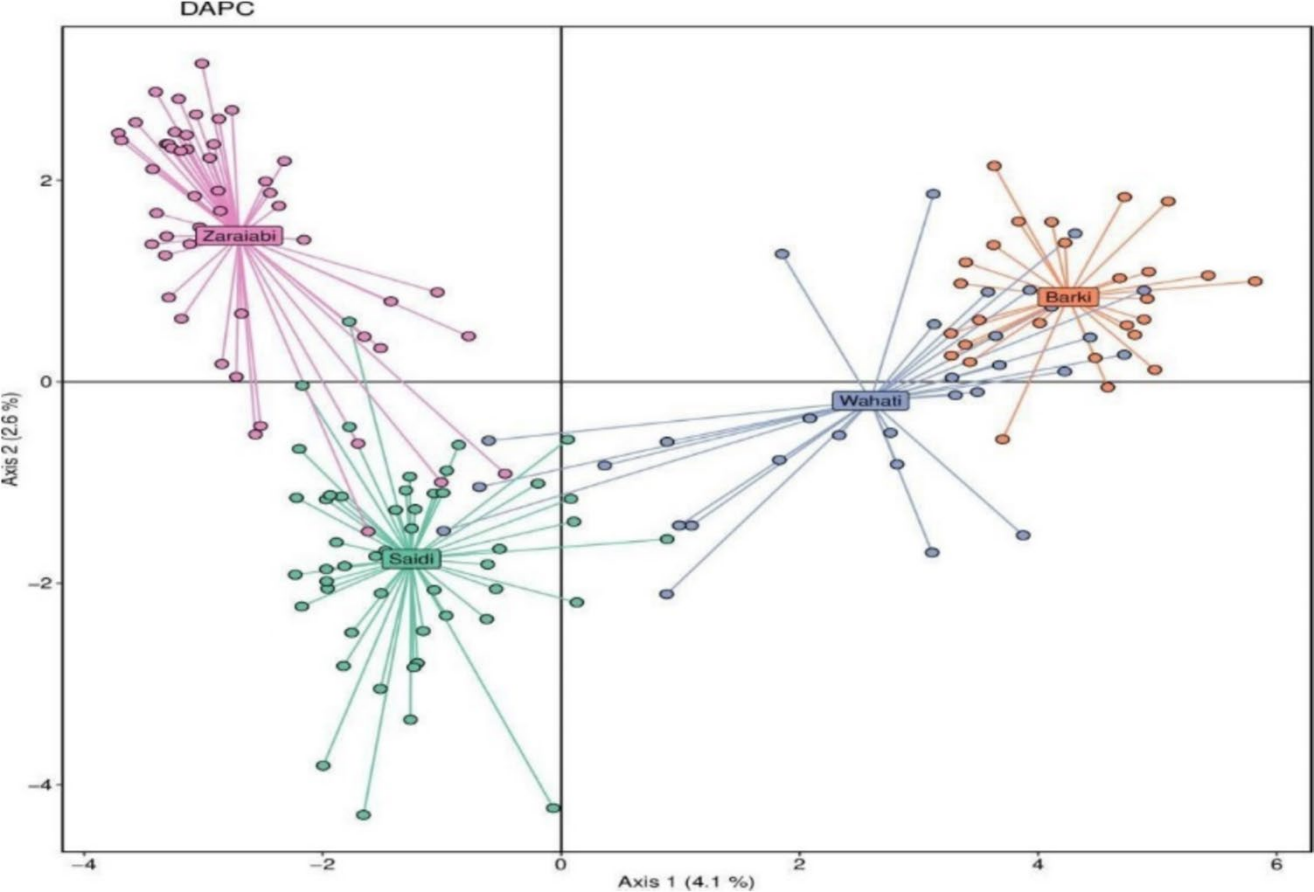
Discriminant analysis Principal Components (DAPC). Each breed (Zaraibi, Barki, Wahati and Seidi) organized in a circle with a connecting line (Aboul-Naga et al. 2023)

Phylogenetic analyses play an important role in unraveling the genetic correlation of the Zaraibi goats with other goat populations, shedding light on their evolutionary history and genetic relatedness. In this context, Aboul-Naga et al. (2023) conducted a comprehensive study revealing significant levels of genetic diversity and differentiation among Egyptian goat populations. Utilizing clustering analysis, Bayesian Information Criterion (BIC), Principal Component Analysis (PCA) and Structure Analysis, they identified distinct groups and patterns of admixture among local goat breeds. Their findings (Fig. 2) demonstrated that the Zaraibi goats exhibit remarkable genetic separation from other local goat breeds, highlighting their unique genetic makeup and distinctiveness within the Egyptian goat population. This significant separation underscores the importance of the Zaraibi breed in the context of local genetic diversity and conservation efforts.

The clear genetic distinctiveness of Zaraibi goats suggests that they have followed a unique evolutionary path, potentially shaped by geographical isolation, environmental adaptation, and human-mediated selection. As a result, the Zaraibi breed represents a valuable genetic resource for breeding programs to improveproductivity, and adaptation to local environmental conditions. Their conservation is therefore essential to preserving Egypt’s livestock biodiversity.

## Challenges, opportunities and prospects for improving Zaraibi Goats

### Challenges

Improving the productivity and sustainability of Zaraibi goats requires tackling key challenges while leveraging opportunities for genetic and management improvements. A comprehensive approach that integrates scientific advancements, strategic breeding programs, and effective management practices is essential for ensuring long-term success. The key challenges include:

- *Epigenetics:* Epigenetics presents significant challenges in livestock research and breeding, particularly in identifying epigenetic factors that change across successive generations at cellular level. Methylation patterns are not constant and are influenced by several environmental factors. Longitudinal studies are needed to detect factors influencing methylation patterns and differentiate between causal and consequential epigenetic variations. Transgenerational epigenetic variants can appear in all tissues, and advanced technologies to detect DNA methylation can improve the accuracy and efficiency of the breeding programs.
- *Small herd size:* Goat production in Egypt is predominantly managed by smallholders who typically keep only a few animals as household livestock and rely on village bucks for natural mating. These smallholders rarely maintain records for their animals and are not involved in breeders’ associations or sire testing groups. The lack of structural organization makes it difficult to implement a nationwide selection program to improve its production.
- *Complex relationship between production traits and heat stress (HS) tolerance:* Genomic analysis of Zaraibi goats has identified several candidate genes associated with HS tolerance, many of which also have multiple functions across different biological processes (Aboul-Naga et al. 2023). These include reproduction, feed intake, immune response, metabolism, steroid biosynthesis, cell–cell signaling, cell growth, and stress response. Identifying these genomic loci can help monitor and enhance HS tolerance while improving overall productive performance.
- *Lack of public awareness:* There is a general lack of public awareness regarding the economic benefits of animal genetic improvement, even among policy makers. This makes it challenging to plan and secure funding for national programs aimed at improving the genetic potential of Zaraibi goats.
- *High costs of genomic analysis:* The high cost associated with genomic analysis presents a barrier for researchers and breeding programs organizer in developing countries. Additionally, donor organizations are often reluctant to fund long-term breeding programs. There is a need for dedicated national funds to support such crucial initiatives.
- *Climate changes:* Evidence clearly indicates that heat and drought will be major factors affecting animal production in hot, dry areas over the next 50 years. Furthermore, it is widely acknowledged that Egypt is one of the country’s most vulnerable to climate change, with negative impacts on animal performance and the livelihoods of vulnerable rural communities.
- *Government policies and strategies:* Small ruminants, especially goats, have low priority in the government policies and strategies despite their significant socioeconomic role in the livelihoods of rural community, particularly for poor families and women.

### Opportunities

Despite the challenges facing the improvement of Zaraibi goats, there are numerous opportunities to enhance their productivity and resilience. The unique characteristics of Zaraibi goats offer significant potential for genetic improvement, better management practices, and increased market value. By recognizing and utilizing these opportunities, breeders and researchers can make substantial progress in enhancing the breed’s overall performance and adaptability. Key opportunities for improving Zaraibi goats include:

- *High genetic diversity: *The high genetic diversity within Zaraibi goat populations provides a valuable opportunity to identify elite animals for economically important traits. This diversity can be utilized to enhance breed performance through well-structured selection and breeding programs.
- *Positive genetic gain*: Given the currently underdeveloped state of the Zaraibi population, significant genetic gains can be achieved through well-planned selection programs. This presents a promising opportunity for improving the breed’s productivity and efficiency.
- *Strong family and community relationships*: The close-knit family and community structure among smallholders can be harnessed for community-based breeding programs. Collective breeding efforts can lead to more sustainable genetic improvements and better herd management.
- *Customer preference for local products*: The strong preference of local consumers to traditional meat and dairy products presents a significant advantage. This demand can drive efforts to enhance the breed’s productivity while ensuring a steady supply of high-quality local products.
- *Adaptation to harsh conditions*: Zaraibi goats are well-adapted to the harsh environmental conditions across different regions of Egypt. Their resilience makes them a promising genetic resource for maintaining and improving productivity under challenging conditions.
- *Socioeconomic importance*: Zaraibi goats play a crucial socioeconomic role as household dairy animals, particularly for women and children in marginal areas with limited feed resources. This highlights the importance of supporting the breed through targeted breeding and management programs.

### Prospects

A well-structured breeding program is essential for sustainably improving Zaraibi goats and ensuring their resilience. The program should integrate genetic selection, data recording, and stakeholder involvement while securing financial and policy support. The primary goals could be to improve productive performance, preserve genetic diversity, and support rural communities reliant on these goats.

A successful breeding program begins with comprehensive data recording. Farmers and breeding organizations should systematically track key traits such as milk yield, growth rate, reproductive efficiency, and disease resistance. Standardized recording schemes and farmer training will ensure consistency, while a centralized database will facilitate accurate genetic evaluations and informed breeding decisions.

Genetic evaluation and selection should combine traditional methods (phenotype and pedigree) with genomic selection for more accurate predictions and early identification of superior animals. To expand the selection base, an open nucleus breeding scheme should be implemented, where high-performing bucks from a central breeding nucleus are distributed to farmers. This strategy enhances genetic improvement while minimizing inbreeding. The program’s effectiveness depends on strong stakeholder involvement. Farmers, researchers, policymakers, and industry representatives must collaborate to define breeding goals, improve husbandry, and ensure knowledge transfer. Training programs and breeder cooperatives will further facilitate these efforts. Sustained funding is crucial for successful genetic evaluation and implementation of breeding program. In this regard, government and private investment should support breeding infrastructure, performance recording, and farmer training. Development organizations must recognize Zaraibi goats’ socioeconomic value and allocate resources accordingly. Additionally, financial incentives or microfinance programs can encourage small-scale farmers to participate in structured breeding programs.

Beyond breeding, market development will enhance economic returns. Expanding the goat cheese market, especially through dairy processing units managed by women, can increase income for smallholders and breeders. Support of marketing infrastructure, branding, and certification of premium dairy products can further boost profitability.

By implementing these strategies, Zaraibi goats can be sustainably improved, ensuring their long-term role in food security, rural livelihoods, and agricultural resilience.

## Conclusions

The present comprehensive review presents the genetic characteristics, challenges, opportunities, and prospects of the Egyptian Nubian (Zaraibi) goat. By adopting a multidisciplinary approach that integrate genetics, breeding, management, and socioeconomic factors, we gain valuable insights into the complexities and potential of this promising subtropical breed.

Zaraibi goats exhibit remarkable genetic diversity, offering significant opportunities for identifying elite individuals for economically important traits. However, several challenges hinder their full potential, including small herd sizes, limited research on specific genes, and the complex relationship between production traits and heat stress tolerance. Addressing these challenges requires dedicated efforts, particularly through longitudinal studies, community-based breeding programs, and public awareness of the breed’s socioeconomic benefits. Despite these challenges, Zaraibi goats present numerous opportunities for improvement, with positive genetic gains expected from well-structured selection programs. Strong community relationships and consumer preference for local products further underscore the breed’s potential. Leveraging genomic analyses, initiating open nucleus flock programs, and implementing sound management practices are key strategies for enhancing the future of Zaraibi goats.

Looking ahead, it is crucial to prioritize the sustainable improvement and conservation of Zaraibi goats through utilization, recognizing their vital role in rural livelihoods. Development agencies and policymakers should integrate Zaraibi goats into the rural development programs, support breeding initiatives, and promote market development for goat products. Future research efforts should focus on addressing knowledge gaps in epigenetics and relationship with heat stress tolerance.

By embracing these recommendations and fostering collaboration among stakeholders, we can unlock the full potential of Zaraibi goats, ensuring their resilience, productivity, and socioeconomic impact, ultimately securing a brighter future for both the breed and the rural communities that depend on it.

## Data Availability

All data analyzed during the current study are available from the corresponding author on request.
